# Anti-Interleukin-17 Therapy of Severe Psoriatic Patients Results in an Improvement of Serum Lipid and Inflammatory Parameters’ Levels, but Has No Effect on Body Composition Parameters

**DOI:** 10.3390/life11060535

**Published:** 2021-06-09

**Authors:** Éva Anna Piros, Ákos Szabó, Fanni Rencz, Valentin Brodszky, Norbert Wikonkál, Pál Miheller, Miklós Horváth, Péter Holló

**Affiliations:** 1 Department of Dermatology, Venereology and Dermato-Oncology, Semmelweis University, 1085 Budapest, Hungary; wikonkal.norbert@med.semmelweis-univ.hu (N.W.); hollo.peter@med.semmelweis-univ.hu (P.H.); 2 Rácz Károly Doctoral School of Clinical Medicine, Semmelweis University, 1085 Budapest, Hungary; akos.szabo@uni-corvinus.hu; 3 Department of Health Economics, Corvinus University, 1093 Budapest, Hungary; fanni.rencz@uni-corvinus.hu (F.R.); valentin.brodszky@uni-corvinus.hu (V.B.); 4 Military Hospital-State Health Centre, 1134 Budapest, Hungary; 5 1st Department of Surgery and Interventional Gastroenterology, Semmelweis University, 1082 Budapest, Hungary; miheller.pal@med.semmelweis-univ.hu (P.M.); horvath.miklos@med.semmelweis-univ.hu (M.H.)

**Keywords:** psoriasis, body composition, interleukin-17 inhibitor, metabolic syndrome, obesity

## Abstract

BACKGROUND: Psoriasis is frequently accompanied by metabolic syndrome. Effect of anti-tumor necrosis factor therapies on increases in body weight is well-known. Data on the effects of interleukin-17 inhibitors are limited. Authors determined the effect of anti-interleukin-17 therapies on the body composition and serum lipid and inflammatory parameters among severe psoriatic patients. METHODS: Thirty-five severe psoriatic patients were enrolled. Twenty-two received secukinumab and 13 received ixekizumab as their 2nd-or 3rd-line biological treatment. Before treatment initiation and 6 months later, laboratory examinations measuring metabolic and inflammatory panels and body composition analyses were performed. RESULTS: After 6 months, a significant reduction was observed in psoriasis area severity index (*p* < 0.001) from 18 to 0, in c-reactive protein (*p* < 0.001) from 6.6 to 4.00 mg/L, in low-density lipoprotein-cholesterol (*p* = 0.004) from 3.69 to 3.19 mmol/L, and an improvement in high-density lipoprotein-cholesterol (*p* = 0.022) from 1.31 to 1.40 mmol/L. Median baseline body mass index was 32.80 kg/m^2^. The body composition parameters did not show any significant changes. CONCLUSIONS: Anti-interleukin-17 therapy of severe psoriatic patients does not cause significant changes in body composition parameters. Improvements in the lipid and inflammatory parameters might have a beneficial effect on patients’ cardiometabolic status. This effect might be detectable in high-risk obese psoriatic patients.

## 1. Introduction

Psoriasis is a chronic autoinflammatory disorder mediated by T_helper_1 and T_helper_17 (Th1 and Th17) cells, and it affects 2–3% of the population worldwide [1]. The aetiology of the disease is multifactorial, and the combination of a genetic predisposition with environmental trigger factors (drugs, stress, infection, and obesity) is responsible for disease development [2]. Psoriasis primarily affects the skin and the nails. It is common to also affect the joints in the form of psoriatic arthritis [3]. The main cytokines currently known to be involved in disease development are tumour necrosis factor (TNF), interferon-gamma (IFN-γ), interleukin-12 (IL-12), IL17A-F, IL-22, and IL-23 [4].

Severe cases of psoriasis are often accompanied by metabolic syndrome, obesity, diabetes mellitus, and cardiovascular diseases based on shared immunopathogenetic pathways, and these comorbidities may impact the patients’ health-related quality of life and mortality [5]. Thus, untreated severe psoriasis may reduce life expectancy by up to five years [6]. A meta-analysis showed that the prevalence of metabolic syndrome was 2.2 times higher among psoriatic patients than among non-psoriatic controls [7]. Ferdinando et al. studied 97 psoriatic patients for metabolic syndrome and compared them to 97 controls. Higher values of systolic blood pressure, body mass index (BMI), fasting glucose, waist circumference, and angina pectoris frequency and lower levels of high-density lipoprotein-cholesterol (HDL-cholesterol) were measured among psoriatic patients [8]. 

The human body can be divided into two main compartments: body fat mass (BFM) and fat-free mass (FFM). In terms of sex and age, there may be significant individual differences in the distribution of the compartments. Approximately 80% of the body is FFM, which mainly includes visceral and skeletal muscle mass (SMM) and bone tissue, which is approximately 7% and total body water (TBW), which is 60–70%. TBW can be further divided into 30% intracellular water (ICW) and 70% extracellular water (ECW). The ratio of ECW and TBW is an important indicator of body water balance. Body cell mass (BCM) is defined as the sum of cells in the muscles, internal organs, and central nervous system that are active in metabolism [9]. Additional indices can be calculated from parameters specific to fat-free mass, body fat, and muscle mass by using an analogue method similar to that used for calculation of BMI. In these formulas, we can calculate the fat free mass index (FFMI), body fat mass index (BFMI) and skeletal muscle mass index (SMMI) by using the equation of parameter (kg)/height in metres^2^. These indices, which also include the BMI, allow for a comparison of parameters regardless of height. 

In clinical practice, detailed body-composition analyses can be assessed using a bioelectrical impedance analyser (BIA), and they are based on the different electrical conductivities of different tissues. Impedance consists of electric resistance and reactance. Phase angle (PA) describes the phase shift between total voltage and total electric current. A higher PA value corresponds to a higher integrity of the cell membrane. High PA values are most commonly found in healthy individuals with strong cell membranes and high BCM, while lower PA values indicate that cells cannot store energy, and the cell membrane is thinner [10]. BIA examinations are simple, inexpensive, reproducible, and do not require qualified staff. Also, they are based on the use of nonionizing radiation that makes this method suitable for follow-up tests. The only known risk of BIA is that arrhythmia can develop in patients with pacemakers or implantable cardioverter defibrillators. Therefore, the analyses should be conducted with caution in this patient group [11]. 

In this self-controlled study, our aim was to determine the long-term (six-month) effect of novel anti-IL-17 therapies (secukinumab and ixekizumab) on body composition, serum metabolic parameters (fasting glucose, total cholesterol, HDL-cholesterol, low-density lipoprotein-cholesterol (LDL-cholesterol), triglyceride, alanine aminotransferase (ALT), aspartate aminotransferase (AST), gamma glutamyl transferase (GGT)), and serum inflammatory parameter c-reactive protein (CRP). 

## 2. Materials and Methods

This study was conducted in the Department of Dermatology, Venereology, and Dermato-Oncology of Semmelweis University, Budapest, Hungary. Body-composition analyses were performed at the First Department of Surgery and Interventional Gastroenterology of Semmelweis University, Budapest, Hungary. Statistical analyses were assessed at the Department of Health Economics, Corvinus University of Budapest, Hungary. The study was performed between September 2019 and March 2021, and each patient was followed-up for 6 months. Approval was obtained from the Regional Institutional Scientific and Research Committee of Semmelweis University, Budapest, Hungary (licence number: 154/2019, date of approval: 12 September 2019.), and the study meets all the requirements of the Declaration of Helsinki. All enrolled patients were informed about the process of the study and signed patient consent forms. 

### 2.1. Study Design

The effectiveness of the anti-psoriatic therapy was measured by dermatological disease severity and health-related quality of life scales: the psoriasis area severity index (PASI) and dermatology life-quality index (DLQI) [12]. These parameters were assessed twice: before starting treatment and after 6 months. 

We enrolled patients with very severe skin symptoms that greatly decreased their health-related quality of life (PASI > 15 and DLQI > 10). Moreover, patients were included who received ineffective topical and/or systemic therapies for a minimum of 3 months prior to IL-17 treatment. Our patients had several other comorbidities and were receiving other medications not related to psoriasis. Patients’ lifestyle and medications remained unchanged throughout the follow-up period, and no new therapy was allowed. 

During the laboratory examinations, we determined the serum metabolic parameters (fasting glucose, total cholesterol, HDL-cholesterol, LDL-cholesterol, triglyceride, ALT, AST, and GGT) and serum CRP. Among the patients who received antidiabetic therapies, we also measured the haemoglobin A1c% (HbA1c %) levels. Blood samples were taken after 8 h of fasting before therapy initiation and, in a similar fashion, after 6 months of therapy. Laboratory parameters were determined by an autoanalyzer (Beckman Coulter AU 5800 Chemistry Analyzer, Tokyo, Japan) according to the manufacturer’s instructions.

During our study, detailed body-composition analyses were performed to determine the following parameters: body weight, BMI, FFM, soft lean mass, body fat mass (BFM), body fat percent (BFP), SMM, TBW, extracellular (EC) water, intracellular (IC) water, ECW ratio, protein content, mineral content, body cell mass, bone mineral content, visceral fat area, and phase angle. All patients were measured using the same InBody 770 (InBody Co., Ltd. model number: BPM040S12FXX, 15, Heugam-gil, Ipjang-myeon, Seobuk-gu, Cheonan-si, Chungcheongnam-do 331-824, Republic of Korea) bioelectrical impedance analyser after 6 h of fasting before treatment initiation and, in a similar fashion, after 6 months always at the same time. 

Subgroup analyses were also performed as to whether the type of IL-17 inhibitor and previous biologicals have an impact on the results. The generated subgroups are the following: severe psoriatic patients who received ixekizumab therapy, severe psoriatic patients who received secukinumab therapy, severe psoriatic patients who previously received anti-TNF therapy, and severe psoriatic patients who previously received ustekinumab treatment.

### 2.2. Dosing Regimen of Anti-IL-17 Therapies

Secukinumab was administered at the labelled dose, with 300 mg subcutaneously administered in weeks 0, 1, 2, 3, and 4 and then every 4 weeks. Ixekizumab was also administered at the labelled dosing, with 160 mg subcutaneously administered in week 0; 80 mg administered in weeks 2, 4, 6, 8, 10, and 12; and then 80 mg administered every 4 weeks.

### 2.3. Evaluation of Data

Taking into account the small sample size and skewed distribution of data, median values and interquartile ranges (IQRs) of the examined parameters (clinical, laboratory, and body-composition values) were calculated before therapy initiation and after 6 months. Differences between the initial and 6-month follow-up periods were analysed by the nonparametric Wilcoxon signed-rank test. Subgroups of patients were compared by using Mann–Whitney U test. The relationship of baseline and 6-month values was also tested by Spearman's correlations. All analyses were carried out using IBM SPSS Statistics version 25 software. A *p*-value of < 0.05 was considered statistically significant.

## 3. Results

### 3.1. Patients’ Characteristics

Baseline patients’ characteristics can be found in Table 1.

Thirty-five adult patients with severe plaque-type psoriasis were enrolled in the study (21 men and 14 women). At the time of enrolment, the median age of patients was 49 (37–62) years, and the median disease duration time was 22 (13–27) years. Overall, 18 patients (51.42%) from the study population had psoriatic arthritis verified by a rheumatologist. All enrolled patients at the time of enrolment had very severe skin symptoms; the median PASI score was 18.0 (14–30), and the median DLQI score was 17.0 (10–27). Six patients (17.14%) were bionaïve; they had not received any biologic therapies, yet they had received topical corticosteroid and/or methotrexate (MTX) or acitretin treatment. Twenty-nine patients (82.86%) received one of the anti-IL-17 medications, secukinumab or ixekizumab, after being unsuccessfully treated with systemic traditional and biologic therapies. Unsuccessful treatment was determined as primary inefficacy or loss of drug efficacy in the form of worsening the psoriatic skin and joint symptoms. Before changing the treatment regimen, a minimum of three months of ineffective treatment were required. Among the previously used systemic therapies were MTX, acitretin, cyclosporine, TNF blockers (adalimumab, infliximab, etanercept), and IL-12/23 antagonist (ustekinumab). In these cases, anti-IL-17 therapies were introduced as second- or third-line systemic biologic therapies. In this real-world study, the allocation of the applied therapeutic monoclonal antibody was determined by the different physicians who were treating the patients; thus, it was completely random which interleukin-17 inhibitor therapy they were receiving. The allocation was also based on the S3 European and the relevant national guidelines and the access to the current medicinal product. 

The presence of metabolic syndrome occurred in eighteen patients (51.42%), with elevated fasting glucose levels (≥100 mg/dL) in 18 patients (51.42%), low HDL-cholesterol levels (≤40 mg/dL) in 12 patients (34.28%), and elevated triglyceride levels (≥150 mg/dL) in 15 patients (42.85%). Eleven patients (31.42%) were on antihypertensive treatment, 6 patients (17.14%) were on antihypertensive and antidiabetic treatments, 7 patients (20%) were on antihypertensive and statin therapies, and 3 patients (8.57%) were on antihypertensive, antidiabetic and statin treatments. When measured, 21 patients (60%) had elevated CRP (>5 mg/L). 

### 3.2. Clinical and Laboratory Findings

Changes in the clinical and laboratory parameters can be found in Table 2.

Thirteen patients (37.14%) received ixekizumab, and 22 patients (62.86%) received secukinumab therapy. After six months, we observed significant improvement in both skin symptoms and health-related quality of life. The PASI score was reduced (*p* < 0.001) from a median value of 18.00 to a median value of 0.0, and the DLQI value also improved (*p* < 0.001) from a median value of 17.00 to a median value of 0.0. 

A dramatic improvement of skin and joint inflammation, measured by PASI and DLQI scores, was accompanied by significant improvements in CRP and ESR. The median value of CRP was reduced (*p* < 0.001) from 6.60 mg/L to 4.00 mg/L, and the median value of ESR was reduced (*p* = 0.004) from 17.00 mm/h to 7.00 mm/h. Among the metabolic parameters, we observed statistically insignificant improvements in the fasting glucose, total cholesterol, triglyceride, and GGT levels. Transaminases (AST and ALT) remained stable during the six months. The cardioprotective HDL-cholesterol levels significantly improved (*p* = 0.022) from a median value of 1.31 mmol/L to 1.40 mmol/L, and the atherogenic LDL-cholesterol levels significantly decreased (*p* = 0.004) from a median value of 3.69 mmol/L to 3.19 mmol/L after six months of effective anti-IL-17 treatment. Interestingly, of the few patients who suffered from diabetes, HbA1c% levels were statistically significantly worsened. This may be due to diet failure.

### 3.3. Body-Composition Analyses

Body-composition measurements can be seen in Table 3.

Only a small number of patients, 3 of 35, had normal BMI. Thirty-two patients (91.42%) were overweight (BMI > 25.0 kg/m^2^), of which 21 patients (60%) were obese (BMI > 30.0 kg/m^2^). The median baseline body weight was 97.00 (75.70–111.00) kg, and the median baseline BMI was 32.80 (27.14–37.97) kg/m^2^. An elevated (>0.39) ECW ratio was observed in 5 patients (14.28%), while a <7° phase angle was found in 32 patients (91.42%). After six months of effective anti-IL-17 treatments, we did not find any significant differences in the examined body-composition parameters. We observed a slight decrease in the median value of the body weight, body-fat percent, visceral fat area, body cell mass, fat-free mass, FFMI, and SMMI. The median value of the TBW, ECW ratio, phase angle, and total body score remained stable.

### 3.4. Comparing Body Composition and Laboratory Parameters between Subgroups of Patients Treated with Secukinumab or Ixekizumab

We compared the effect of the two drugs on body composition and laboratory parameters. There were no significant differences between secukinumab and ixekizumab therapy in body composition parameters neither at the baseline nor at the sixth month of the applied therapies. In the case of the laboratory parameters, only the baseline ALT was significantly higher in the ixekizumab group with no other significant differences.

### 3.5. Comparing Body-Composition Parameters between Subgroups of Patients Previously Treated with Anti-TNF or Ustekinumab Therapy

In our study population, the anti-tumor necrosis factor or ustekinumab therapy were used before interleukin-17 inhibitor treatment among patients who were not bionaïve. The impact of the previously used therapies on the observed parameters were also statistically analyzed. The number of patients who received anti-TNF, ustekinumab, and systemic non-biological therapies or were bionaïve, respectively, were 8 (22.87%), 15 (42.87%), 6 (17.13%), and 6 (17.13%).

Body composition and laboratory parameters were not influenced by the previously used therapies in our study.

### 3.6. Correlation Analysis of Clinical, Body-Composition, and Laboratory Parameters

Spearman’s test revealed weak correlation between changes of PASI and CRP (0.133). Weak reverse correlation could be detected between changes of CRP and HDL-cholesterol levels (−0.21) (Table 4).

## 4. Discussion

Psoriasis and obesity are connected through the human leukocyte antigen-class I allele (HLA-Cw6) locus and alpha-ketoglutarate-dependent dioxygenase (FTO) gene rs9939609 [13,14]. The common key molecules that link metabolic syndrome and psoriasis are TNF, IL-17, CRP, leptin, and adiponectin [8,15]. IL-17 is one of the central molecules of the Th17/IL-23 axis that interferes with hepatic steatosis in nonalcoholic fatty liver disease (NAFLD), and insulin signalling and IL-17R expression correlate with insulin resistance [16,17]. Forty-seven percent of the psoriatic patients had NAFLD shown by ultrasound, while ratio in the age-sex-BMI adjusted healthy control group of NAFLD was only 28% [18]. IL-17 also increases TNF- and lipopolysaccharide (LPS)-induced IL-6 and C-C chemokine ligand 20 (CCL20) production and upregulates IL-17RA expression in 3T3-L1 adipocytes [19]. In human bone-marrow mesenchymal cells, IL-17A inhibits the differentiation of adipocytes and the expression of adipokines, while in differentiated adipocytes, it amplifies lipolysis. Among obese psoriatic patients, lower levels of leptin and adiponectin can be measured; moreover, a higher PASI score corresponds to a lower adiponectin level [20,21,22]. Systemic inflammation, which causes persisting symptoms of psoriasis, has several indicators, for example, serum level of CRP, HDL-cholesterol, and Apolipoprotein-A-I. Apolipoprotein-A-I concentration is significantly lower in patients with psoriasis compared to non-psoriatic, healthy controls [23]. The concentration of HDL-cholesterol is reciprocally proportional with the systemic inflammation.

During “psoriatic march”, Th17-produced IL-17 cytokines play an important role in the development of psoriasis, insulin resistance, and obesity that may finally lead to atherosclerotic cardiovascular diseases, acute coronary syndrome, and stroke. The latest biologic therapies, such as secukinumab and ixekizumab, target these cytokines. Secukinumab is a fully human monoclonal antibody (mAb), and ixekizumab is a humanized IgG4 mAb. TNF, the other central cytokine in psoriatic development, belongs to the Th1 line. The effect of anti-TNF therapies on body weight is well documented. Adalimumab and infliximab therapies significantly increased body weight and BMI among psoriatic patients [24,25]. Methotrexate therapy, due to its hepatotoxicity, may lead to non-alcoholic fatty liver disease. Therefore, liver transaminases should be monitored closely in these patients [26]. In the presence of obesity, methotrexate therapy should be used with caution [27]. In the case of the effect of other systemic biologic antipsoriatic therapies, Gisondi et al. compared the effect of ustekinumab and infliximab on body weight changes among plaque-type psoriatic patients. After seven months of treatment, infliximab showed a significant increase in mean body weight and BMI compared with patients treated with ustekinumab [28]. In the post-hoc analysis of the phase III VOYAGE 1 and 2 studies, guselkumab showed higher efficacy than adalimumab, and it was consistent across different body-weight groups [29]. In the phase III UltIMMA-1 and 2 studies, rizankizumab showed consistently higher efficacy compared to ustekinumab in all examined body-weight groups [30]. In the case of anti-IL-17 therapies, such data are limited, and their effect on body composition and metabolic parameters are controversial. A post-hoc analysis of FIXTURE-ERASURE-SCULPTURE studies included 3010 patients who were treated with secukinumab, etanercept, or placebo and followed-up for 52 weeks. After 52 weeks, secukinumab reduced the high-sensitivity CRP (hs-CRP), uric acid levels, and BMI values but had no effect on plasma glucose, lipid, and liver enzyme parameters [31]. In another study, waist circumference, BMI, blood pressure, fasting glucose, total cholesterol, HDL-and LDL-cholesterol, triglyceride, ALT, AST, and creatinine levels were determined among psoriatic patients with metabolic syndrome who received methotrexate or secukinumab therapy for 12 months. In the secukinumab group, significant changes were not observed in the parameters, and in the methotrexate group, a significant increase was observed in liver enzymes [32]. In a retrospective study, Wang et al. examined the effect of secukinumab on 99 psoriatic patients during a 24-week treatment and found that hs-CRP was significantly reduced, while body weight and BMI were significantly increased at week 24 [33]. In the UNCOVER studies, significant changes were not observed in body weight or serum lipid and glucose parameters after 60 weeks [34]. 

Our work is a real-life study in which significant improvements were observed in the disease-related dermatological parameters, PASI and DLQI, as well as the inflammatory parameter CRP after six months of anti-IL-17 treatment. In the metabolic panel, statistically significant improvements were not observed for fasting glucose, total cholesterol, triglyceride, and GGT levels, while statistically significant improvements were observed for HDL-cholesterol and LDL-cholesterol levels. This is partly due to the reduction of the generalized inflammation as a consequence of the strong anti-inflammatory effect of interleukin-17 inhibitors and partly due to the direct effect of the monoclonal antibody. In mouse experiments, treatment with anti-interleukin-17A antibody significantly reversed the effect of imiquimod-induced changes in the liver biomarkers related to injury as well as protein/lipid metabolism; however, treatment with interleukin-17A further aggravated them through the NF-kB pathway [35,36]. HDL-cholesterol and albumin levels were improved in the anti-interleukin-17 treated group. 

Although it is a limited comparison, data from the FIXTURE-ERASURE-SCULPTURE, the UNCOVER, and two retrospective studies of Wang et al. and Gisondi et al. show that lower values of baseline total cholesterol levels and higher values of baseline triglyceride levels were measured than in our study population. In the case of the baseline HDL-cholesterol levels, values higher than ours were only found in the UNCOVER studies. The highest baseline LDL-cholesterol values were measured in our study compared to the others [31,32,33,34]. Moreover, 60% of our study population was obese. In the previously mentioned UNCOVER studies and retrospective Wang et al. study, the rate of obese individuals was less than 8% and almost 40% of the study population. The lower prevalence of obese patients might have influenced the laboratory findings. In our study population, 27 patients (77.14%) had >100 cm^2^ visceral fat area, and the maximum was 292.8 cm^2^. Psoriasis is associated with metabolic syndrome and obesity, and this association is stronger with increasing disease severity. Based on Langan et al.’s findings in a population-based study in the United Kingdom, metabolic syndrome was seen in 32% of patients with mild disease (BSA ≤ 2%), in 36% of patients with moderate disease (BSA 3–10%), and in 40% of patients with severe disease (BSA > 10%). The risk of obesity in psoriatic patients increases with the severity of the skin disease: a 14% increased chance of obesity was found with mild psoriasis, a 34% increased chance of obesity was found with moderate psoriasis, and a 66% increased chance of obesity was found with severe psoriasis [37]. There is a clear association with abdominal obesity; with increasing degree of abdominal obesity, the risk of having major cardiac event, stroke, or myocardial infarct also increases [38]. Based on the 1995 WHO report in our study population, 13 female patients (37.14%) had >35% BFP, and 18 male patients (51.42%) had >25% BFP [39]. The average PA value in the healthy white population is approximately 7 degrees [10]. A total of 91.42% of our study population had PA values less than 7°. After six months of anti-IL-17 treatment, we did not find any significant changes in the body-composition parameters. Comparing the two different IL-17 inhibitor therapies, there was no significant difference on body-composition and laboratory parameters. No significant impact of previously used systemic biologic therapies could be observed in body-composition and laboratory parameters during subgroup analysis. 

## 5. Conclusions

IL-17 inhibitor therapy in severe psoriatic patients does not lead to significant changes in weight or body-composition parameters. However, improvements that we have seen in lipid and inflammatory parameters during anti-IL-17 treatment might have a beneficial effect on comorbidities, especially on the patients’ cardiometabolic status. This may be more meaningful in severe psoriatic patients who also have metabolic syndrome. These patients could be identified as a group with higher cardiovascular risk.

Interdisciplinary collaborations with internists are required for appropriate patient care. Regarding the efficacy of IL17 inhibitor biologicals, further investigations should be performed on high-risk obese patient groups with metabolic syndrome. 

## 6. Study Limitations

Because of the low patient number, patients were not stratified according to their comorbidities; diabetes mellitus, hypertension, dyslipidaemia, or metabolic syndrome may influence treatment efficacy, body-composition, and laboratory parameters. Our patients had several other comorbidities and were receiving other medications not related to psoriasis, which is another limitation of the study; however, the patients’ lifestyles and medications remained unchanged throughout the follow-up period. Although our subgroup analysis did not reveal it, the previous systemic therapies and their interruption may have an influence on the observed parameters. Last, this study was a self-controlled study in which the patients were their own controls. 

## Figures and Tables

**Table 1:** Descriptive characteristics of severe psoriatic patients treated with interleukin-17 inhibitors.

Patients’ Comorbidities	N (%) or Median (IQR)
**Total sample**	35 (100)
**Sex**	
Women	14 (40)
Men	21 (60)
**Age (years)**	49 (37–62)
**Disease duration (years)**	22 (13–27)
**BMI (kg/m^2^)**	32.8 (27.14–37.97)
**Psoriatic arthritis**	18 (51.42)
**Treatments**	
Bio-naive	6 (17.14)
Secukinumab	22 (62.86)
Ixekizumab	13 (37.14)

BMI, body mass index; IQR, interquartile range.

**Table 2:** Changes in clinical and laboratory parameters of severe psoriatic patients treated with interleukin-17 inhibitors.

Characteristics	Baseline, Median (IQR)	6-Month Follow-Up, Median (IQR)	*p*-Value (Wilcoxon Signed-Rank Test)
**Clinical Characteristics**			
PASI (0–72)	18.00 (14–30)	0.0 (0–4)	<0.001
DLQI (0–30)	17.00 (10–27)	0.0 (0–1)	<0.001
**Laboratory Characteristics**			
CRP (mg/L)	6.60 (3.20–15.00)	4.00 (2.10–5.60)	<0.001
ESR (mm/h)	17.00 (5–37)	7.00 (4–22)	0.004
HDL-cholesterol (mmol/L)	1.31 (1.16–1.53)	1.40 (1.25–1.60)	0.022
LDL-cholesterol (mmol/L)	3.69 (2.75–4.24)	3.19 (2.27–3.83)	0.004
Total cholesterol (mmol/L)	5.40 (4.40–6.50)	5.26 (4.30–6.20)	0.161
Triglyceride (mmol/L)	1.52 (1.11–2.00)	1.50 (1.10–2.38)	0.116
Fasting glucose (mmol/L)	5.60 (4.80–6.30)	5.22 (4.70–6.40)	0.640
AST (U/L)	24.00 (19–35)	24.00 (18–28)	0.207
ALT (U/L)	24.00 (18–43)	24.00 (17–34)	0.011
GGT (U/L)	31.00 (21–65)	28.00 (20–60)	0.120

PASI, psoriasis area severity index; DLQI, dermatology life-quality index; CRP, c-reactive protein; ESR, erythrocyte sedimentation rate; HDL-cholesterol, high-density lipoprotein-cholesterol; LDL-cholesterol, low-density lipoprotein-cholesterol; AST, aspartate aminotransferase; ALT, alanine aminotransferase; GGT, gamma glutamyl transferase.

**Table 3:** Body-composition parameters of severe psoriatic patients treated with interleukin-17 inhibitors.

Characteristics	Baseline Median (IQR)	6-Month Follow-Up Median (IQR)	*p*-Value (Wilcoxon Signed-Rank Test)
Body weight (kg)	97.00 (75.70–111.00)	96.70 (77.40–112.20)	0.269
BMI (kg/m^2^)	32.80 (27.14–37.97)	32.80 (27.21–38.18)	0.439
Fat-free mass (kg)	55.20 (49.60–69.70)	54.60 (48.6–70.60)	0.895
FFMI (kg/m^2^)	20.17 (18.76–21.88)	20.00 (18.32–22.46)	0.631
TBW (L)	40.60 (36.40–51.10)	40.60 (36.20–51.80)	1.000
Protein (kg)	10.80 (9.70–13.70)	10.70 (9.40–14.00)	0.741
Minerals (kg)	3.67 (3.09–4.71)	3.61 (3.25–4.75)	0.405
Body fat mass (kg)	33.30 (23.10–47.20)	33.30 (24.20–46.70)	0.548
BFMI (kg/m^2^)	10.99 (7.87–17.22)	11.15 (8.08–17.86)	0.605
Body fat percent (%)	37.10 (28.90–47.60)	36.90 (29.00–47.30)	0.885
Skeletal muscle mass (kg)	30.80 (27.30–39.10)	30.40 (26.40–40.50)	0.727
SMMI (kg/m^2^)	11.21 (10.30–12.11)	11.09 (10.15–12.61)	0.556
ECW ratio	0.38 (0.38–0.39)	0.38 (0.38–0.39)	0.984
Phase Angle (◦)	5.60 (5.00–6.00)	5.60 (5.30–6.10)	0.214
Visceral fat area (cm^2^)	165.50 (115.20–229.20)	164.20 (110.70–220.80)	0.524
Body cell mass (kg)	36.0 (32.20–45.20)	35.60 (31.20–46.60)	0.741
Bone mineral content (kg)	3.06 (2.48–3.86)	2.95 (2.583.91)	0.561
ECW (L)	15.70 (13.90–19.80)	15.70 (13.90–19.50)	0.698
ICW (L)	25.10 (22.50–31.50)	24.90 (21.80–32.60)	0.709
InBody Score	65.0 (53.0–72.0)	65.0 (54.0–73.0)	0.777

BMI, body mass index; FFMI, fat-free mass index; TBW, total body water; BFMI, body fat mass index; SMMI, skeletal muscle mass index; ECW ratio, extracellular water ratio; ECW, extracellular water; ICW, intracellular water.

**Table 4:** Spearman’s correlations between changes of clinical outcome and laboratory parameters.

	PASI (0–72) Change (N = 35)	CRP (mg/L) Change (N = 35)	ESR (mm/h) Change (N = 35)	HDL-cholesterol (mmol/L) Change (N = 35)	LDL-cholesterol (mmol/L) Change (N = 33)
PASI (0–72) Change (N = 35)	-	0.133	0.006	−0.031	0.046
CRP (mg/L) Change(N = 35)	-	-	0.424 *	−0.210	0.054
ESR (mm/h) Change(N = 35)	-	-	-	−0.001	−0.007
HDL-cholesterol (mmol/L) Change (N = 35)	-	-	-	-	−0.180
LDL-cholesterol (mmol/L) Change (N = 33)	-	-	-	-	-

PASI, psoriasis area severity index; CRP, c-reactive protein; ESR, erythrocyte sedimentation rate; ESR, erythrocyte sedimentation rate; HDL-cholesterol, high-density lipoprotein-cholesterol; LDL-cholesterol, low-density lipoprotein-cholesterol; * *p* < 0.05.

## Data Availability

The data presented in this study are available on request from the corresponding author. The data are not publicly available in accordance with consent provided by participants on the use of confidential data.

## Abbreviations

Th	T helper
TNF	tumour necrosis factor
IFN-γ	interferon gamma
IL	interleukin
BMI	body mass index
HDL-cholesterol	high-density lipoprotein-cholesterol
BFM	body fat mass
FFM	fat-free mass
SMM	skeletal muscle mass
TBW	total body water
ICW	intracellular water
ECW	extracellular water
BCM	body cell mass
FFMI	fat-free mass index
BFMI	body fat mass index
SMMI	skeletal muscle mass index
BIA	bioelectrical impedance analyzer
PA	phase angle
LDL-cholesterol	low-density lipoprotein-cholesterol
ALT	alanine aminotransferase
AST	aspartate aminotransferase
GGT	gamma glutamyl transferase
CRP	c-reactive protein
ESR	erythrocyte sedimentation rate
PASI	psoriasis area severity index
DLQI	dermatology life-quality index
HbA1c%	haemoglobin A1c%
BFP	body fat percent
IQR	interquartile ranges
MTX	methotrexate
FTO gene rs9939609	alpha-ketoglutarate-dependent dioxygenase gene rs9939609 variant
NAFLD	non-alcholic fatty liver disease
IL-17R	interleukin-17 receptor
LPS	lipopolysaccharide
CCL	CC chemokine ligand
mAb	monoclonal antibody
VOYAGE 1 study	A study of guselkumab in the treatment of participants with moderate to severe plaque-type psoriasis
VOYAGE 2 study	A study of guselkumab in the treatment of participants with moderate to severe plaque-type psoriasis with randomized withdrawal and retreatment
UltIMMA-1 study	BI 655066 (risankizumab) compared to placebo and active comparator (ustekinumab) in patients with moderate to severe chronic plaque psoriasis
UltIMMA-2 study	BI 655066 versus placebo and active comparator (ustekinumab) in patients with moderate to severe chronic plaque psoriasis
FIXTURE	Safety and efficacy of secukinumab compared to etanercept in subjects With moderate to severe, chronic, plaque-type psoriasis
ERASURE	Efficacy and safety of subcutaneous secukinumab for moderate to severe chronic plaque-type psoriasis for up to 1 year
SCULPTURE	Efficacy and safety of subcutaneous secukinumab (AIN457) for moderate to severe chronic plaque-type psoriasis assessing different doses and dose regimens
hs-CRP	high-sensitivity c-reactive protein
UNCOVER-1,2	A phase 3 study in participants with moderate to severe psoriasis
UNCOVER-3	A study in participants with moderate to severe psoriasis
NF-kB	nuclear factor kappa light chain enhancer of activated B cells
BSA	body surface area
WHO	World Health Organisation
